# Deep learning driven silicon wafer defect segmentation and classification

**DOI:** 10.1016/j.mex.2025.103158

**Published:** 2025-01-06

**Authors:** Rohan Ingle, Aniket K. Shahade, Mayur Gaikwad, Shruti Patil

**Affiliations:** Symbiosis Institute of Technology, Pune Campus, Symbiosis International (Deemed University), Pune, Maharashtra, India

**Keywords:** Silicon wafers, Integrated circuit, Defect segmentation, Deep learning, Wafer defects, Quality management, Image segmentation, Wafer Defect Segmentation and classification using Deep Learning

## Abstract

Integrated Circuits are made of various transistors that are embedded on a silicon wafer, these wafers are difficult to process and hence are prone to defects. Defecting these defects manually is a time consuming and labour-intensive task and hence automation is necessary. Deep Learning approach is better suited in this case as it is able to generalize defects if trained properly and can be a solution to segmentation and classification of defects automatically. The segmentation model mentioned in this study achieved a Mean Absolute Error (MAE) of 0.0036, a Root Mean Squared Error (RMSE) of 0.0576, a Dice Index (DSC) of 0.7731, and an Intersection over Union (IoU) of 0.6590. The classification model achieved 0.9705 Accuracy, 0.9678 Precision, 0.9705 Recall, and 0.9676 F1 Score. In order to make this process a more interactive, an LLM with Q&A capabilities was integrated to solve any doubts and answer any questions regarding defects in wafers.

This approach helps automate the detection process thus improving quality of end product.

• Successful and precise defect segmentation and classification using Deep Learning was achieved.
• High-intensity regions after post-processing.
• An LLM offering defect analysis and guidance was streamlined.

## Graphical abstract

**Figure:**
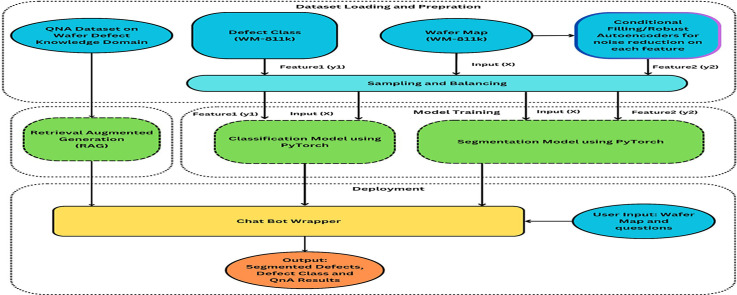

Specifications table

**Table:** 

Subject area:	Computer Science
More specific subject area:	Deep Learning
Name of your method:	Wafer Defect Segmentation and classification using Deep Learning
Name and reference of original method:	None
Resource availability:	WM-811k Dataset (Kaggle) https://www.kaggle.com/datasets/qingyi/wm811k-wafer-map

## Background

The modern world is always evolving and hence in order to improve efficiency and automate repetitive tasks a solution is required. For this, various machines are developed [[1], [2], [3]]. A computer in one of the machines that play a vital role in improving productivity in various fields. However, building a computer is a very difficult and challenging task, the processor is one of the most difficult components to manufacture and it is one of the most important components in a computer [3,4].

Processors are made up of millions or even billions of transistors embedded on a silicon wafer and is called a “die” [4]. This die is the most critical and hardest to produce part of the processor. These dies are created in batches on silicon wafers which are prone to defects at different stages of production [1,3]. These defects can affect the quality and reliability of the processors made from them. Hence, making it very important to address and mitigate them effectively to ensure high-quality processors are made [1,3,5].

Manual inspection for each silicon wafer is both time consuming and labour intensive. Hence, to improve efficiency we need ways to automate the process [3,5]. Defects on wafers can also point to issues with the manufacturing process [3]. Detecting and addressing these defects is important for improving individual chips and also for maintaining consistent quality over time [3,5].

Implementing large language models (LLMs) in the process helps in establishing interactive systems that can help detect, understand, and mitigate these defects in the wafers [2,6,7]. This can significantly improve efficiency and maintain the high standards required for modern processors [2,6,7].

## Method details

In this section we will discuss about the dataset, annotation generation, model architecture of segmentation model and classification model and training process.

### Dataset

The WM-811K dataset is a classification dataset comprising wafer maps without any pixel-level defect annotations [8]. Each wafer map represents a silicon wafer with various potential defects. The dataset lacks ground truth annotations for defects, necessitating the generation of pixel-wise annotations for subsequent analysis [8]. The dataset consists of wafer maps where defected areas are marked with an intensity value of "1." However, these defect signals are often scattered across the maps, introducing significant noise that complicates accurate detection and analysis [3].

Table 1, which summarizes defect labels and their corresponding descriptions:

**Table 1:** Defect Labels and Descriptions.

Label	Description
Loc	Localized defects in a specific region of the wafer.
Edge-Loc	Defects concentrated along the wafer's edge.
Center	Defects primarily found at the center of the wafer.
Edge-Ring	Ring-shaped defects near the wafer's edge.
Scratch	Line-shaped defects caused by physical scratching.
Random	Randomly distributed defects without a clear pattern.
Near-full	Defects covering most of the wafer area.
Donut	Circular defects resembling a donut shape.
No Defect	Wafers with no visible defects.
Unknown/Unlabelled	Defects that are unlabelled or not classified.

Below is Table 2, which provides an overview of the number of wafer map entries:

**Table 2:** Overview of Wafer Map Entries.

Category	Number of Entries	Description
Total Entries	811,457	Total number of wafer maps analyzed.
Labelled Entries	172,950	Wafer maps with specific defect labels.
Unlabelled Entries	638,507	Remaining entries without specific defect annotations.

**Fig. 1:**
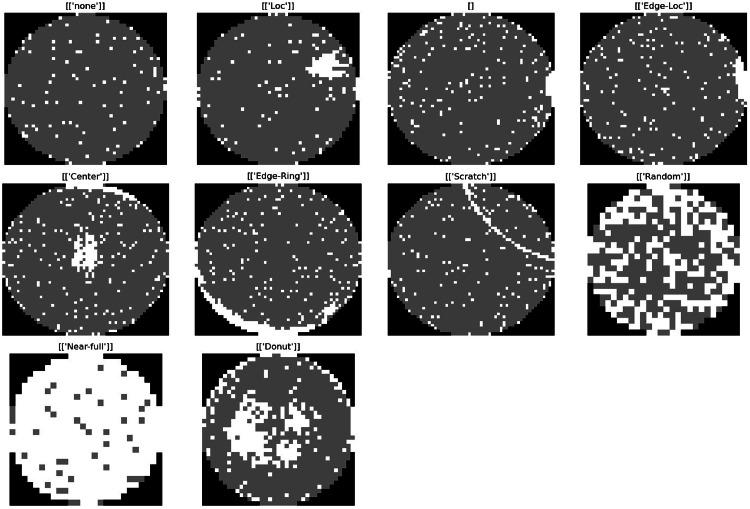
Visualization of Defects included in WM-811K Dataset.

Below is Fig. 1, which illustrates the visualization of defects included in the WM-811K dataset:

### Annotation Generation, Sharpening and Balancing

First, we will start by applying conditional filling to the wafer maps using a 3 × 3 window and logic to consolidate defect signals where if at least 3 surrounding pixels also had defect intensity. This will help us remove major noise from the dataset as preprocessing step.

Next, we will use a type of autoencoders called “Robust Deep Autoencoder” to remove any remaining noise, but we will only use “Huber loss” as loss function [9]. The autoencoder was trained to reconstruct defect-free wafer maps, highlighting discrepancies between the input and reconstructed maps. These differences isolated defect regions, transforming pre-processed maps into representations where clusters of defects became more distinct.

Huber Loss is Given as:

$$L_{\delta }\left(a\right)=\left\{ \begin{array}{c} \frac{1}{2}{\left(y_{i}-\hat{y}i\right)}^{2},\;if\;\left|y_{i}-\hat{y}i\right|\leq \delta \\ \delta .\left|y_{i}-\hat{y}i\right|-\frac{1}{2}\delta^{2},\;if\;\left|y_{i}-\hat{y}i\right|>\delta \end{array}\right.$$

The dataset has been balanced to ensure equitable representation of defect types, resulting in a total of 30,519 samples across nine classes. The most common defect, Edge-Ring, has 9680 samples, while the majority class, No Defect, was down sampled to 6000 samples. Other defects, including Edge-Loc (5189), Center (4294), and Loc (3593), are well represented, whereas rarer defects like Scratch (1193), Random (866), Donut (555), and Near-Full (149) are included in smaller proportions. This balanced sampling strategy improves fairness and allows the model to effectively learn distinguishing features, even for less frequent defect types.

After autoencoder reconstruction, additional post-processing was applied using a noise-removal filter to further refine defect maps by eliminating residual isolated noise. This combination of conditional filling, autoencoder processing, and post-processing yielded well-defined defect clusters, enhancing annotation quality and reducing the impact of noise. By employing resizing with “INTER_AREA” interpolation and refining defect maps through this pipeline, the dataset was optimally prepared for robust segmentation and classification.

**Fig. 2:**
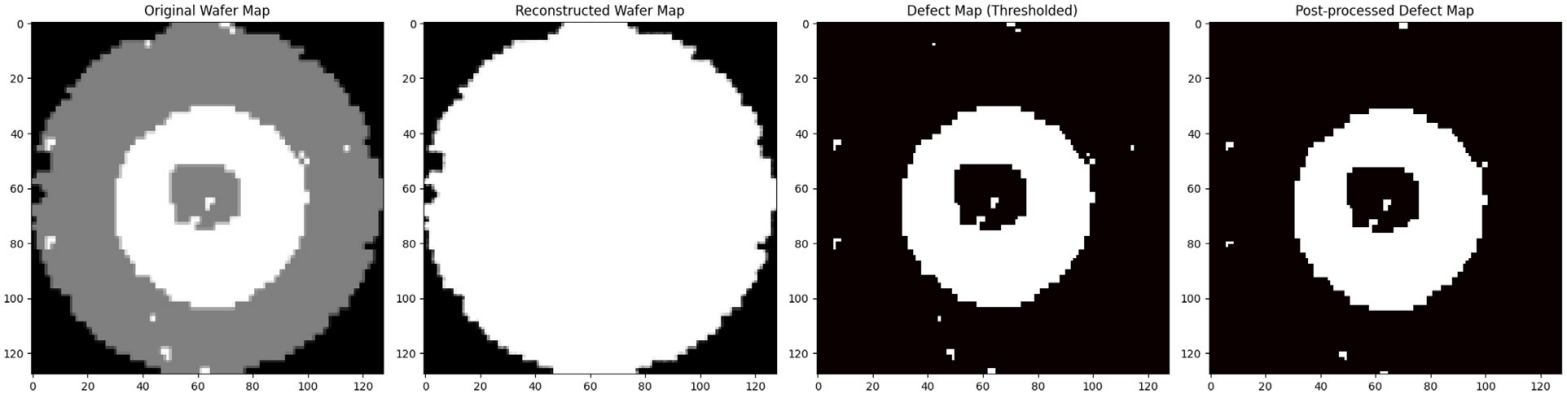
Pre-processing Progress and Final Processed Defect Map.

This Fig. 2 highlights the preprocessing steps, with the "Post-Processed Defect Map" serving as the final pixel-level annotation.

### Deep Learning Segmentation Model using PyTorch

A custom deep learning segmentation model was developed using the PyTorch framework [10,11] to accurately identify and segment defects in wafer maps. The architecture comprises two main components: an encoder and a segmentation decoder, designed to efficiently extract and reconstruct feature representations necessary for precise defect localization [1,3,5].

### Model Architecture Segmentation Model

**Fig. 3:**
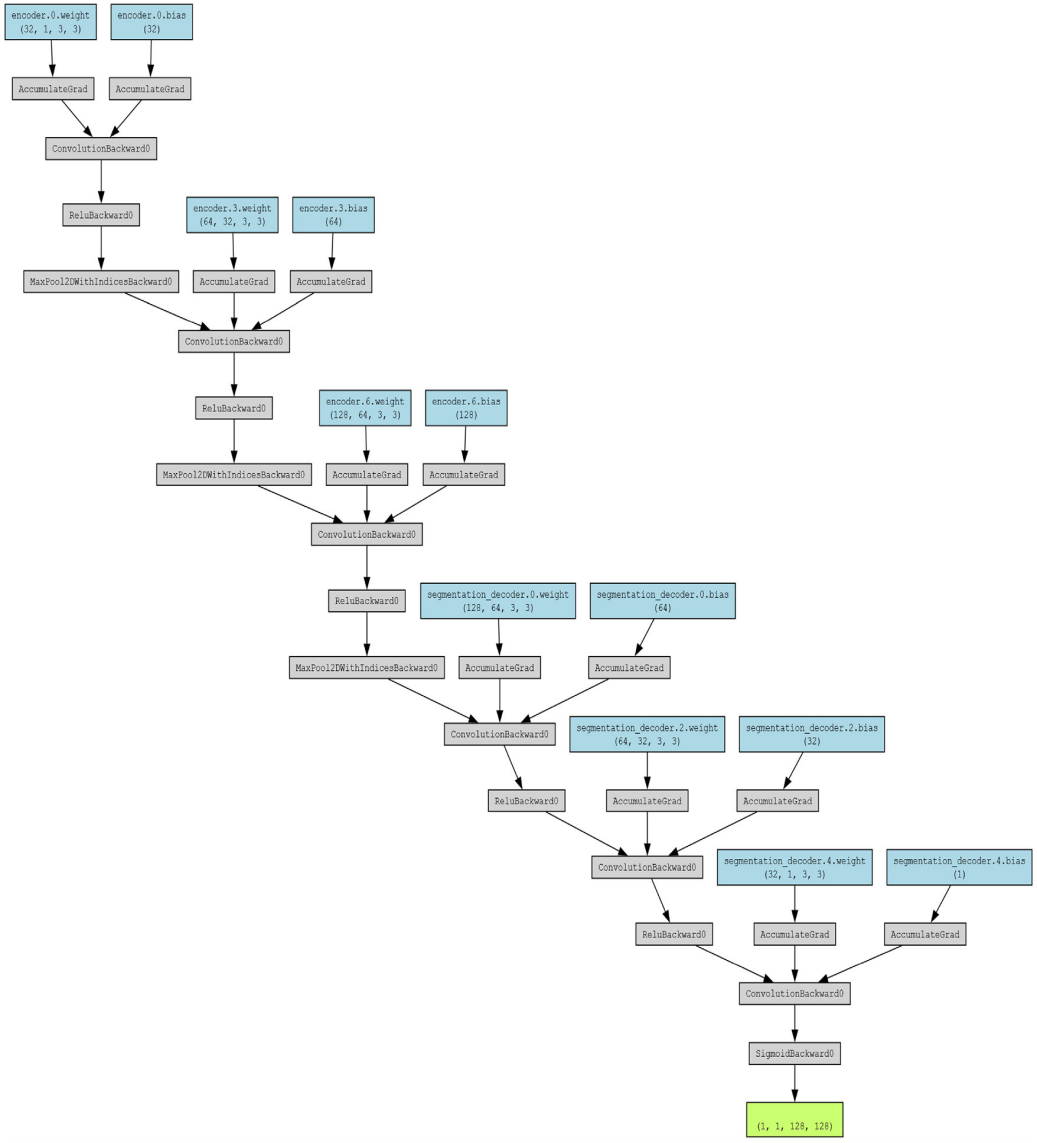
Model architecture of Deep Learning Segmentation Model.

This Fig. 3 depicts the architecture of the deep learning model used for defect segmentation.

**Encoder:** The encoder consists of a sequential stack of convolutional and pooling layers aimed at progressively reducing the spatial dimensions of the input while increasing feature abstraction ([3,11]). Specifically, the encoder includes three convolutional blocks as mentioned in Table 3:

**Table 3:** Encoder Architecture - Convolutional Blocks (Segmentation Model).

Convolutional Block	Layer	Details
First Block	Convolutional Layer	32 filters, kernel size: 3, stride: 1, padding: 1
	Activation Function	ReLU
	Pooling Layer	MaxPooling, kernel size: 2, stride: 2
Second Block	Convolutional Layer	64 filters, kernel size: 3, stride: 1, padding: 1
	Activation Function	ReLU
	Pooling Layer	MaxPooling, kernel size: 2, stride: 2
Third Block	Convolutional Layer	128 filters, kernel size: 3, stride: 1, padding: 1
	Activation Function	ReLU
	Pooling Layer	MaxPooling, kernel size: 2, stride: 2

This structure enables the encoder to capture hierarchical features from the wafer maps, effectively condensing spatial information into a compact representation suitable for defect segmentation ([3,5]).

**Segmentation Decoder:** The segmentation decoder mirrors the encoder's architecture through a series of transposed convolutional layers, which serve to upsample the encoded feature maps back to the original input resolution (3,11]). The decoder comprises three transposed convolutional blocks as represented in Table 4:

**Table 4:** Decoder Architecture - Transposed Convolutional Blocks.

Transposed Convolutional Block	Layer	Details
First Block	Transposed Convolutional Layer	64 filters, kernel size: 3, stride: 2, padding: 1, output padding: 1
	Activation Function	ReLU
Second Block	Transposed Convolutional Layer	32 filters, kernel size: 3, stride: 2, padding: 1, output padding: 1
	Activation Function	ReLU
Third Block	Transposed Convolutional Layer	1 filter, kernel size: 3, stride: 2, padding: 1, output padding: 1
	Activation Function	Sigmoid

The final transposed convolutional layer employs a sigmoid activation function to generate a binary segmentation mask, delineating defect regions within the wafer maps ([1,3]).

**Training Procedure:** The model was trained using pre-processed wafer maps and their corresponding post-processed defect masks. The dataset was partitioned into training and testing subsets with an 80:20 split to facilitate robust evaluation. The training parameters were as follows:

• Loss Function: Binary Cross-Entropy Loss (BCELoss)
  $$Loss\;Function=\;-\frac{1}{N}\sum_{i=1}^{N}\left[y_{i}\cdot \log\left(p_{i}\right)+\left(1-y_{i}\right)\cdot \log\left(1-p_{i}\right)\right]$$
• Optimizer: The Adam optimizer was selected with a learning rate of 0.001 to efficiently navigate the loss landscape.
• Batch Size: A batch size of 32 was employed to balance computational efficiency and gradient estimation accuracy.
• Epochs: 20.
• Early Stopping: Implemented with patience = 5 to prevent overfitting.

### Deep Learning Classification Model using PyTorch

The classification model based on deep learning was developed with the help of PyTorch library in Python [11] to automate the classification of defects in silicon wafers. The model consists of various layers including convolutional encoder with fully connected layers in order to extract various latent features and classify the wafer map [1,3,5].

### Model Architecture Classification Model

**Fig. 4:**
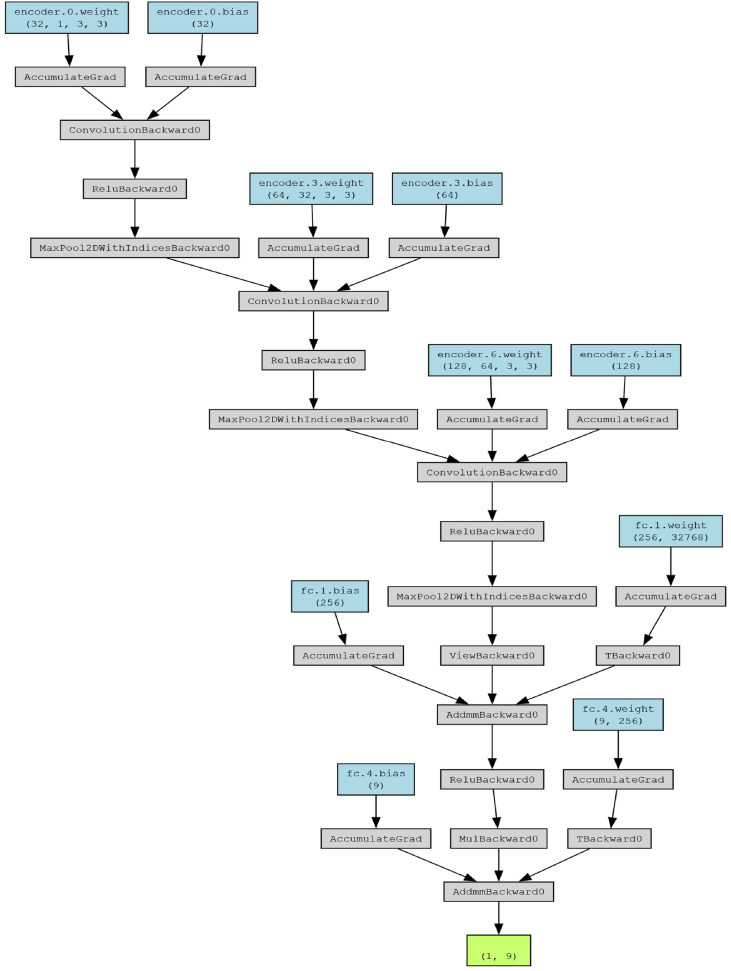
Model architecture of Deep Learning based Classification Model.

Fig. 4 illustrates the model architecture of the Deep Learning Classification Model, showcasing a multi-layered network structure designed to effectively classify input data through hierarchical feature extraction and reconstruction.

**Encoder:** The encoder is responsible for extracting hierarchical features from the input wafer maps. It comprises a sequence of convolutional and pooling layers organized into three convolutional blocks mentioned in Table 5:

**Table 5:** Encoder Architecture - Convolutional Blocks (Classification Model).

Convolutional Block	Layer	Details
First Block	Convolutional Layer	32 filters, kernel size: 3, stride: 1, padding: 1
	Activation Function	ReLU
	Pooling Layer	MaxPooling, kernel size: 2, stride: 2
Second Block	Convolutional Layer	64 filters, kernel size: 3, stride: 1, padding: 1
	Activation Function	ReLU
	Pooling Layer	MaxPooling, kernel size: 2, stride: 2
Third Block	Convolutional Layer	128 filters, kernel size: 3, stride: 1, padding: 1
	Activation Function	ReLU
	Pooling Layer	MaxPooling, kernel size: 2, stride: 2

**Fully Connected Classifier:** the extracted features from the encoder is passed through the hidden layer/fully connected layer that performs the classification task:

1. Flattening Layer:
  I. Converts the 2D feature maps into a 1D feature vector, enabling input to fully connected layers [11].
2. First Fully Connected Layer:
  I. Linear Layer: Transforms the flattened feature vector into 256 neurons.
  II. Activation Function: ReLU, selected for its efficiency in propagating gradients and preventing vanishing gradient issues [10,12].
  III. Dropout Layer: Dropout probability of 0.5 to reduce overfitting, promoting generalization during training [10].
3. Second Fully Connected Layer:
  I. Linear Layer: Maps the 256 neurons to the number of defect classes (9 classes), where each neuron corresponds to one class [1,3].

The final layer outputs logits corresponding to each defect class, which are processed by a softmax function during inference to calculate class probabilities [11].

**Training Procedure:** The classification model was trained using the pre-processed and balanced wafer maps dataset. The dataset is divided into training and testing subsets with an 80% and 20% size respectively. To evaluate model on unseen data i.e. testing data to check accuracy of the model in real life cases.

1. Loss Function: Cross Entropy Loss.
2. Optimizer: Adam optimizer was selected with a learning rate of 0.001.
3. Batch Size: 16.
4. Epochs: 20.
5. Early Stopping: Implemented with patience = 5.

### LLM for Question and Answering

A chatbot is implemented with the help of advanced Natural Language Processing techniques to serve the purpose of Q&A related to the defects in silicon wafers. This method uses the classification model to interpret the kind of defect in the wafer and implements Retrieval Augmented Generation as a source data to generate relevant answers to the questions [[13], [14], [15]].

### Dataset Creation

The dataset used for Q&A chatbot is scraped using ChatGPT's web search abilities. The scraped dataset along with data available in public domain is augmented and structured in format suitable for Retrieval Augmented Generation with the help of LLMs [2,3,7,13].

### Model Architecture

LLaMA 3.2:1b model integrated with Retrieval Augmented Generation is used to make the chatbot ([3,11]). The chatbot uses the classification model to interpret the type of defect withing a given wafer and uses it to give relevant answers [7,15].

### Classification Integration

I. If a wafer map is passed to the model the classification model is used to classify the defect.
II. This defect type is the passed to the model as context which is used to give relevant answers to the asked questions.

### Retrieval Augmented Generation (RAG) using LangChain

I. LangChain Framework: The LangChain library was employed to facilitate the integration of RAG, providing a seamless pipeline for retrieval and generation tasks [[14],[6]].
II. Retrieval System: A vector-based retrieval system is implemented using Chroma [[14]].
III. Embedding Generation: OllamaEmbeddings is used.
IV. RAG Mechanism: When suerr provides the chatbot with a wafer map, it is classified using the classification model and the defect type is passed as context to the model, then the model accepts user query and uses Retrieval Augmented Generation to answer questions with relevant information [[13],[14],[6]].

## Method validation

### Robust Deep Autoencoder

**Fig. 5:**
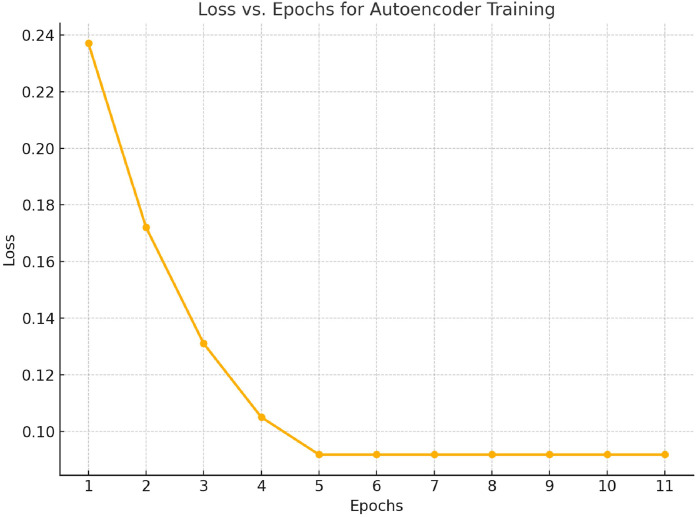
Graph denoting change in Loss over number of epochs while training Robust Deep Autoencoder.

The progression of loss during the autoencoder training from the 1st to the 11th epoch demonstrates a clear learning trend. Initially, the loss exhibits a rapid decrease, dropping from 0.237 in the first epoch to 0.0918 by the fifth epoch. This sharp decline indicates effective learning and substantial improvement in the model's ability to reconstruct input data during the early stages of training. Beyond the fifth epoch, the loss stabilizes at 0.0918, signifying that the model has reached a point of convergence Fig. 5.

### Segmentation Model

Table 6 presents the evaluation metrics used for assessing the performance of the Deep Learning Segmentation Model, including quantitative measures that ensure a comprehensive analysis of segmentation accuracy and effectiveness.

**Table 6:** Evaluation Metrics for Deep Learning based Segmentation Model.

Metric	Value
Mean Absolute Error (MAE)	0.0036
Root Mean Squared Error (RMSE)	0.0576
Signal-to-Noise Ratio (SNR)	2.6914 dB
Dice Index (DSC)	0.7731
Intersection over Union (IoU)	0.6590

The segmentation model is evaluated on various metrics given below:

#### Mean Absolute Error (MAE)

MAE is calculated by flattening the target image and segmented image, then the average deviation between them is calculated. The MAE for our model is 0.0036 which signifies that the predicted images are very close to the ground truth.

$$\frac{1}{n}\sum_{n=1}^{n}\left|y_{i}-\hat{y}i\right|$$

#### Root Mean Squared Error (RMSE)

RMSE captures the standard deviation of the prediction errors. An RMSE value of 0.0576 indicates minimal discrepancies between the model's forecasts and the true values, reflecting high accuracy in the segmentation task.

$$\sqrt{\frac{1}{n}\sum_{i=1}^{n}{\left(yi-{\hat{y}}_{i}\right)}^{2}}$$

#### Signal-to-Noise Ratio (SNR)

SNR, expressed in decibels (dB), provides a ratio of signal intensity (correct predictions) to noise level (errors). The observed SNR of 2.6914 dB suggests there is room for reducing noise. Generally, higher SNR values correspond to stronger alignment between predicted and actual values.

$$10\cdot \log_{10}\left(\frac{P_{signal}}{P_{noise}}\right)$$

#### Dice Index (DSC)

The DSC measures the overlap between segmented predictions and the actual ground truth. With a Dice index of 0.7731, the model demonstrates good segmentation quality. As the DSC approaches 1, the overlap increases, indicating superior segmentation performance.

$$\frac{2\left|P\cap G\right|}{\left|P\right|+\left|G\right|}$$

#### Intersection over Union (IoU)

Also known as the Jaccard Index, IoU compares the union area of the predicted segmentation and the ground truth to their intersection area. A value of 0.6590 represents a reasonably good match, but higher IoU values would reflect further improvement in segmentation accuracy.

$$\frac{P\cap G}{P\cup G}$$

### Classification Model comparative results

Table 7 outlines the evaluation metrics utilized for the Deep Learning Classification Model, providing key measures to analyze its performance in accurately predicting class labels.

**Table 7:** Evaluation Metrics for Deep Learning Classification Model.

Metric	Proposed Model	Reference Model [1]	Reference Model [5]
Accuracy	0.9705	0.9375	0.977 (Multiclass)
Precision	0.9678	0.9381	-
Recall	0.9705	0.9379	-
F1 Score	0.9676	0.9376	-

**Fig. 6:**
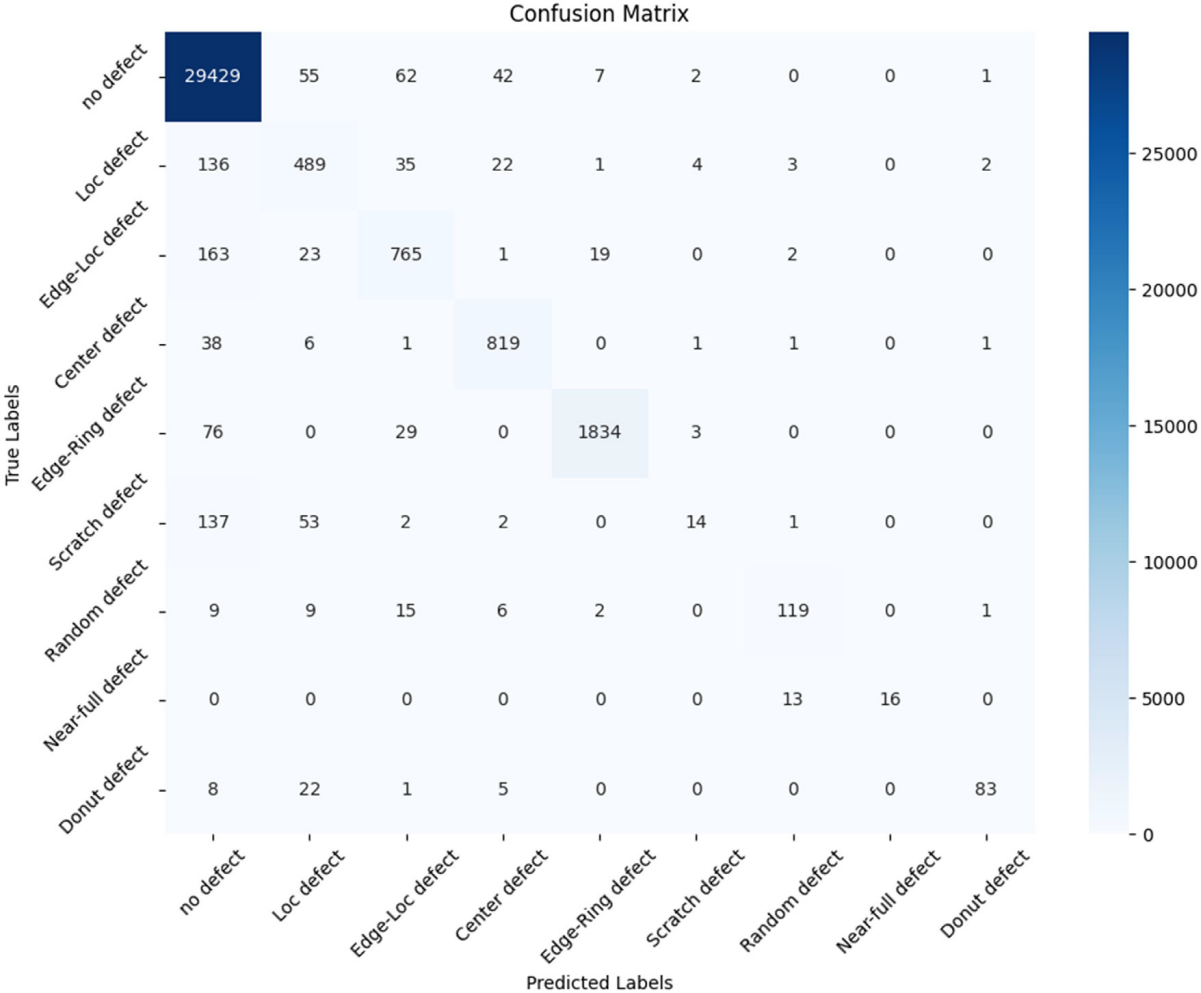
Confusion Matrix for Classification Model.

**Fig. 7:**
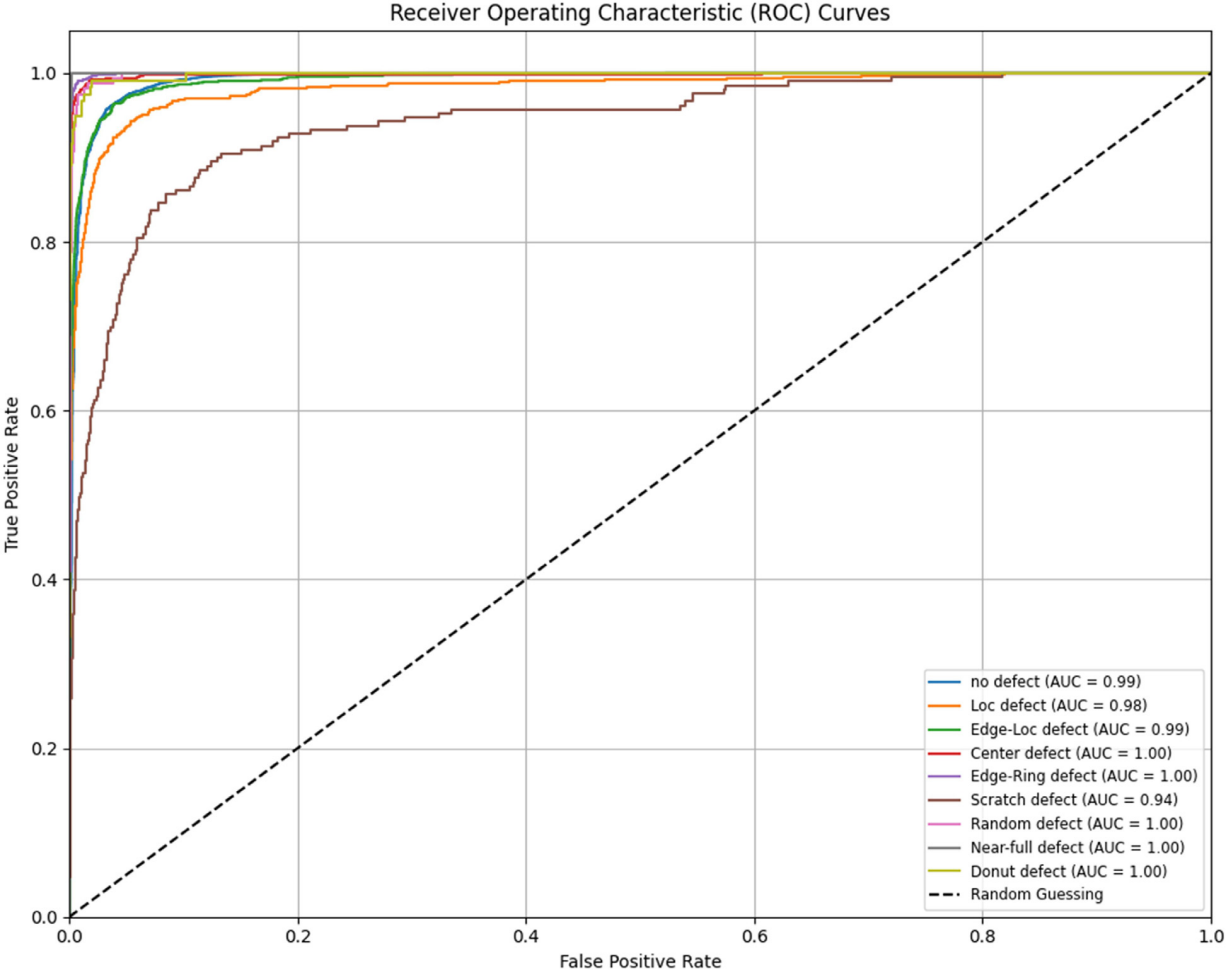
AUC-ROC for Classification Model.

Fig. 6 displays the confusion matrix, showcasing the model's prediction accuracy and error distribution. Fig. 7 illustrates the AUC-ROC curve, highlighting the model's ability to distinguish between classes.

To evaluate the effectiveness of the proposed classification model, four key performance metrics were employed: **Accuracy, Precision, Recall**, and **F1 Score**. Each metric provides a unique perspective on the model's ability to correctly identify and classify wafer defects [[1], [5], [16], [17]].

Accuracy (0.9705)

Accuracy measures the proportion of total predictions that are correct, indicating how often the model produces the right classification overall. A high Accuracy of 0.9705 signifies that the model correctly classifies most samples.

Precision (0.9678)

Precision quantifies the fraction of positively predicted cases that are actually positive. In defect detection, a Precision of 0.9678 means the model has a strong ability to pinpoint true defects, minimizing false alarms.

Recall (0.9705)

Recall represents the ratio of correctly identified positive cases to all actual positive cases in the dataset. A Recall of 0.9705 confirms that the model identifies most real defects, ensuring few defects remain undetected.

F1 Score (0.9676)

The F1 Score is the harmonic mean of Precision and Recall, offering a balanced assessment of the model's overall detection performance. An F1 Score of 0.9676 illustrates the model's consistent reliability in classifying defects with both accuracy and coverage.

## Limitations

Resizing is not lossless, so some information may be lost in the process. In addition, high computation power is required, which can be challenging for systems with limited resources. More data is needed to achieve better results; however, even with extensive datasets, noise reduction is not perfect. Moreover, using a generalized loss reduction method (such as an autoencoder with conditional filling) on resized images of different sizes can cause fine details like very thin scratches in high-resolution wafer maps to be mistaken for noise. Also, this method cannot classify multiple defects within a single wafer map.

## Ethics statements

Not applicable

## CRediT author statement

**Rohan Ingle:** Conceptualization, Methodology, Software, Validity tests, Data curation, preparation, Visualization, Investigation, Supervision, Validation, Writing - Original draft. **Aniket K. Shahade:** Supervision, Review & editing. **Mayur Gaikwad:** Supervision & Review. **Shruti Patil:** Supervision & Review.

## Data Availability

Data will be made available on request.
